# An examination of task factors that influence the associative memory deficit in aging

**DOI:** 10.3389/fpsyg.2022.991371

**Published:** 2022-09-23

**Authors:** Ricarda Endemann, Siri-Maria Kamp

**Affiliations:** Department of Psychology, University of Trier, Trier, Germany

**Keywords:** cognitive aging, episodic memory, associative deficit, strategy, stimulus material, delay

## Abstract

Aging is accompanied by a decline in associative memory, whereas item memory remains relatively stable compared to young adults. This age-related associative deficit is well replicated, but its mechanisms and influencing factors during learning are still largely unclear. In the present study, we examined mediators of the age-related associative deficit, including encoding intentionality, strategy instructions, the timing of the memory test (immediate vs. 24 h delayed) and the material being learned (words vs. pictures) in a within-subject design. Older and younger adults performed seven encoding tasks on word pairs and picture pairs on two consecutive days, followed by item and associative recognition tests. The associative deficit was evident after all encoding tasks. We found no evidence for a difference in the magnitude of the associative deficit between incidental vs. intentional learning conditions. However, there was some evidence for a larger associative memory deficit with pictures versus words when the encoding task was held equal. Sentence generation and interactive imagery instructions in which participants generated their own mediators reduced the magnitude of the associative deficit. However, increased encoding guidance through the provision of mediators did not lead to an alleviation of the deficit, potentially because the specified mediators were implausible or difficult for the older adults to reconcile with prior knowledge. Finally, we found some evidence for a reduced age-related associative deficit with a test delay of 24 h. These results contribute to a better understanding of the factors affecting the relative difficulty of older adults with encoding and retrieving novel associations.

## Introduction

Aging is typically associated with reduced performance in episodic memory (Spencer and Raz, 1995; Old and Naveh-Benjamin, 2008; Park and Reuter-Lorenz, 2009). Kausler and Lair (1965) reported the first evidence that, within episodic memory, older adults have significant problems remembering contextual information, compared to younger adults. By contrast, memory for individual pieces of information from an episodic event is relatively unaffected by aging (Spencer and Raz, 1995). In four experiments, Naveh-Benjamin (2000) demonstrated this dissociation, manipulating stimulus material (word – non-word pairs, word – font pairs), instructions (with a focus to encode items vs. association), and the type of memory test. Consistently, older adults showed strongly reduced associative, but relatively preserved item memory. This age-related associative deficit is now known to be a robust phenomenon, but its magnitude may vary depending on factors like the type of association tested, the stimulus material, encoding instructions or the test format (Old and Naveh-Benjamin, 2008). Another factor may be age differences in the effective use of strategies, because instructing an effective strategy can mitigate the deficit (e.g., Dunlosky and Hertzog, 1998; Bastin et al., 2013; Kamp, 2020).

Mediators that may influence the magnitude of the age-related associative deficit are to date not sufficiently understood. Thus, much of what we know about such mediators comes from meta-analytic examinations, which mostly includes between-subjects comparisons. Only few studies have examined the influence of multiple factors on the associative deficit within experiments and within subjects. Here, we examined in a within subjects design, whether and under which circumstances the encoding mode (incidental vs. intentional), material type (words vs. pictures), test delay (immediate vs. 24 h delayed) and strategy instructions varying in levels of guidance (free strategy selection vs. strategy instruction with self-generated mediators vs. strategy instruction with provided mediators) affect the magnitude of the age-related associative deficit.

### Incidental versus intentional encoding

A factor that can have a strong influence on learning is whether individuals expect that they will be subsequently tested (Hasher and Zacks, 1979). Expecting a memory test typically leads to a more elaborated and thus deeper processing (Craik and Lockhart, 1972). In contrast, incidental learning is associated with lower cognitive load, learning is more casual and automatic (Hasher and Zacks, 1979), and tends to result in shallower processing of the material to be learned (Craik and Lockhart, 1972).

Some evidence suggests that the age-related associative deficit is more prominent in intentional versus incidental encoding tasks (Old and Naveh-Benjamin, 2008). This is, however, only the case with relatively low demands during retrieval, such as in recognition tasks (Spencer and Raz, 1995). For example, in one study older adults performed generally worse under incidental encoding instructions, regardless of whether item or associative memory is involved, resulting in a reduced relative age-related associative memory deficit, whereas an age-related associative deficit was prominent under intentional conditions (Naveh-Benjamin et al., 2009). In contrast, other studies have shown that the age-related association deficit can also be found under incidental learning conditions (Chalfonte and Johnson, 1996; Troyer et al., 2006; Saverino et al., 2016). For instance, Troyer et al. (2006), examined age differences in name or face recognition (item memory) vs. a name-face matching test (associative memory). In three experiments, subjects were instructed to learn names and faces for a later memory test, or not to learn them explicitly but to answer questions about the face, the name, or both. Regardless of age, intentional and deep incidental processing led to positive effects on associative recognition memory, and this effect did not differ by age.

Due to these ambiguous findings, the present study investigated whether the age-related associative deficit is influenced by encoding intentionality when the encoding task is held constant.

### The role of strategy

Depth of processing, relying on working memory processes, in particular maintenance and speed of processing (Salthouse, 1994; Bartsch et al., 2019), plays a crucial role in building an episodic memory trace and is influenced by the strategies that individuals use during encoding. Deeper processing requires more cognitive resources and tends to be reduced in older age (Simon, 1979), potentially due to age-related shrinkage of the prefrontal cortex (PFC), which impairs working memory processes and executive functions (West, 1996; Wagner et al., 1998; Park and Reuter-Lorenz, 2009; Shing et al., 2010). Older adults are thought to employ less strategic elaboration and organization, and linking material to pre-existing knowledge during encoding (Craik and Lockhart, 1972; Shing et al., 2010). Accordingly, the age-related deficit may in part be attributed to a lack of self-initiated strategic processes, binding between items, and deep information processing by creating semantic links (Craik and Lockhart, 1972; Chalfonte and Johnson, 1996; Craik and Rose, 2012).

Supporting the idea that older adults are less likely to engage effective encoding strategies, instructing older subjects to use such strategies can alleviate the associative deficit (Naveh-Benjamin et al., 2007; Dulas and Duarte, 2013; Kamp, 2020). Other studies have reported that older adults are equally likely to report spontaneously using strategies like sentence generation or interactive imagery when they encode two items, but may not use the strategies in the same effective vein as their younger counterparts (Dunlosky and Hertzog, 1998). It is hence unclear to what extent strategy provision can help alleviate the associative deficit, and what characteristics a provided strategy should have in order to effectively do so.

Craik and Rose (2012) suggest that older adults have particularly great difficulty with tasks that require self-initiated processing and relatively less difficulty when more external guidance is available. Encoding instructions thus may differ in the amount of external guidance that they provide. A suitable encoding strategy that provides strong external guidance during memory encoding likely requires a sense of purpose for deeper processing to establish a semantic link, but also sufficient time and attention. Thus, to our knowledge, the effect of the specific level of guidance that the instruction of a specific encoding strategy (such as sentence generation or interactive imagery) provides on the magnitude of the age-related associative deficit has not been studied directly.

### Material type – Verbal versus non-verbal

Another factor that can affect cognitive processes is the type of material to be memorized. Thus, different mechanisms have been found to support the processing of words, objects and faces (Paivio and Csapo, 1973; Bruce and Humphreys, 1994; Rossion et al., 2003). Within the domain of episodic memory, pictures tend to lead to better memory (Paivio and Csapo, 1973). An explanation of this “picture superiority effect” is that processing of pictures operates both semantically and pictorially, which leads to a deeper processing by activating two separate but interconnected pathways (Paivio and Csapo, 1973; Snodgrass and Asiaghi, 1977). Principally, the picture superiority effect seems to be independent of age when individual items must be recalled (e.g., Paivio and Csapo, 1973; Winograd et al., 1982; Maisto and Queen, 1992) or recognized (Ally et al., 2008, 2009). Maisto and Queen (1992) compared older and younger adults with respect to recall of words, pictures, or pictures and their labels. A picture superiority effect was found in both age groups, however, older adults generally performed worse than younger subjects both for single stimuli (pictures or words) and associations (pictures and their labels).

In general, verbal skills tend to remain intact in older adulthood, whereas other abilities such as perceptual speed or spatial orienting decline (Baltes et al., 1999; Park and Reuter-Lorenz, 2009). Relying on this intact verbal processing, older adults may have relatively few difficulties establishing links between words, implying that the associative deficit could be enhanced with pictures. Generally in line with this idea, in a recent study from our group, in which pairs of visually complex pictures were learned, we found neural evidence that older adults tend to focus more on perceptual rather than semantic item features during picture pair encoding (Kamp et al., 2022). Specifically, a “subsequent memory effect” (SME) in the event-related potential, with a posterior scalp distribution and a reversed polarity, was prominent during item processing in the older adults and was negatively correlated with associative memory performance. Assuming that this SME indexes relatively low-level perceptual encoding, this suggests that a lower inclination of older adults to deeply and elaboratively encode pictures may prevent them from effectively forming associations between pictorial stimuli.

In contrary to these ideas, the meta-analysis by Old and Naveh-Benjamin (2008) did not reveal any evidence for differences between verbal and non-verbal material in the magnitude of the associative deficit. Notably, very few studies have contrasted item and associative memory between words and pictures in young and older adults within participants. Ratcliff and McKoon (2015) found a main effect for material type, such that pictures were better remembered than words, but the age-related associative deficit appeared independent of stimulus material. In another study by Guez and Lev (2016), a picture superiority effect was found for item, but not for associative memory, but again, the age-related associative deficit was not modulated by modality. Taken together, the role of stimulus modality, especially in dependence on task instructions, remains unclear.

### Test delay

Another factor that typically has a large impact on memory performance is the delay between study and test; particularly interference within this delay plays a decisive role with regard to forgetting (Underwood, 1957).

In the Rey Auditory Verbal Learning Task, age differences in performance have been shown not only with immediate testing, but to be stable at test delays of 15 min (Carlesimo et al., 1997) to 1 day (Davis et al., 2003). Similarly, for pictures in a picture naming task, recognition performance has been reported to decline in the same vein for older and younger adults over the retention intervals of 1, 7, and 21 days (Mitchell et al., 1990). Other studies showed a drop in performance after immediate recall of single pictures (line drawings) in both age groups (Rybarczyk et al., 1987; Park et al., 1988). Taken together, item memory appears to be similarly affected by test delay in young and older adults.

Few studies have examined the effect of test delay on item or associative memory. In a recent study by Ritchey et al. (2015), younger subjects learned nouns embedded in sentences for two consecutive days. Recognition memory performance was tested on day 2 for all nouns (item memory) and sentences (associative memory) and showed a similar decline due to the delay in item and associative memory performance. Regarding the effects of test delay on the age-related associative deficit, Kuhlmann et al. (2021) reported that in a continuous item-associative recognition task with different intervening lags, item memory was more strongly affected by (especially short) delays than associative memory both for young and for older adults. The associative deficit, however, seemed to be independent on test delay and interference. While relatively short test delays within an experimental session were tested in the latter study, the present study examined the effect of a 24 h delay on item and associative memory in young and older adults.

Taken together, there is a scarcity of prior systematic studies investigating the influence of test delay on the age-related associative memory deficit. Notably, the picture superiority effect can be diminished in delayed retrieval (Rajaram and Pereira-Pasarin, 2007), suggesting that an examination of the effects test delay on item and associative memory in young and older adults will benefit from taking into account the nature of the stimulus material.

### The present study

In the present study, older and younger adults completed several pair encoding tasks, which differed in their instructions during encoding. The first and second task involved a comparative pleasantness judgment under incidental and intentional instructions, respectively. In the third task, participants were free to use any encoding strategy. Item and associative recognition tests for the first three tasks were completed both immediately and 24 h later. In a second session, effective learning strategies were instructed (i.e., sentence generation or interactive imagery), for which specific mediators were provided (high level of guidance; tasks 4 and 5), or for which participants had to generate their own mediators (lower level of guidance, tasks 6 and 7).

In line with many prior studies demonstrating a relative age-related associative memory deficit, we hypothesized that in all tasks, compared to the younger subjects, older adults would show a substantial reduction in associative memory, but an attenuated age-related reduction in item memory. Furthermore, we examined whether this associative deficit is affected by the stimulus material. Secondly, in line with the small body of prior evidence, we hypothesized an enhanced age-related associative deficit under intentional compared to incidental learning conditions. This pattern could be observed due to the younger adults actively engaging deeper, more elaborate encoding processes when the subsequent test is announced, especially benefiting their associative memory, while older adults potentially fail to self-initiate such processes to the same extent (Naveh-Benjamin et al., 2007; Shing et al., 2010; Craik and Rose, 2012). Accordingly, and thirdly, the associative deficit should be mitigated by the provision of effective encoding strategies. In particular, when strategy use was more strongly guided by the provision of specific mediators for each study pair, we expected the associative deficit to be most strongly attenuated. Finally, due to the robustness of the phenomenon in the prior literature, we predicted that the age-related associative memory deficit would be stable across a 24 h delay, although due to the limited amount of prior evidence, this hypothesis was somewhat tentative.

## Materials and methods

All procedures were reviewed and approved by the local ethics committee before data collection began. All participants provided their written informed consent. The data were collected between May and July 2021, at the end of the second lockdown due to the Corona-pandemic in Germany.

### Participants

Older adults (60 years or older) were recruited within a period of 3 months from existing databases and through distributing flyers. The sample of young adults (40 years or younger) was recruited concurrently through a university database and through flyers. The subjects received an Amazon voucher for their participation.

Eighty-eight older (60–86 years) and eighty-nine younger (20–38 years) adults agreed to participate. Due to technical requirements, twelve older and 13 younger subjects could not partake in the study. One older and two younger participants were excluded from all analyses because their memory performance (Pr-score) deviated by more than 2 SD from their age group mean in more than 3 memory measures (item and associative memory measures for each task). Therefore, the final sample contained 75 older adults [age (*M* = 68.17; *SD* = 6.44), sex (37 male/38 female); education (years: *M* = 11.5; *SD* = 1.99)], and 74 young adults [age (*M* = 25.54; *SD* = 5.1), sex (24 male/50 female); education (years: *M* = 12.4; *SD* = 1.1)]. With a desired power of 1-β = 0.95 and α = 0.05, this sample is sensitive to detect an age group × memory type (item vs. associative memory) interaction with a small to medium effect size of *f* = 0.15 (Faul et al., 2007). Two younger and five older participants were unable to participate on the second day due to technical problems, so that the analyses of these tasks included 70 older and 72 young adults.

All older participants were screened with the Telephone Interview for Cognitive Status (Brandt et al., 1988) and showed no abnormalities (all TICS scores > 26).

### Stimulus selection

The stimuli for the memory tasks consisted of 270 words and 270 pictures.

Words were selected from the WWN database (Lahl et al., 2009) with a low to medium arousal level (*M* = 5.6; *SD* = 1.4) and medium (i.e., relatively neutral) valence (*M* = 3.1; *SD* = 1.4). They were combined randomly into 135 word pairs and 5 lists of 27 word pairs each were created, which were randomly assigned to each of the five tasks that included word pairs. These five lists were assigned to different tasks in three versions of the experiment. One version was randomly selected for each subject.

The pictures were taken from the gray-scaled version of the MultiPic database (Duñabeitia et al., 2018). Only pictures that could be matched to a simple word in their representation, using the object label from the WWN database, were included. If an object name duplicated a word from the stimulus list, we randomly selected whether the word or the picture was replaced by another stimulus. The pictures were assembled into 135 picture pairs and divided into five lists, which were assigned to different tasks in the three versions of the experiment.

The words and object labels did not differ in concreteness (words: *M* = 8.84, *SD* = 0.61; pictures: *M* = 8.81, *SD* = 0.50), length (words: *M* = 5.55, *SD* = 1.52; pictures: *M* = 5.43, *SD* = 1.51) or frequency (words: *M* = 3606.89, *SD* = 8026.09; pictures: *M* = 3832.16, *SD* = 7647.1) (all *t*-tests *p* > 0.05).

All word and picture pairs were composed of two different categories (e.g., animal and instrument) and were rated in terms of their semantic relatedness by seven independent persons from the research team. In case a pair was judged as related by at least one rater, the pairs were rearranged, such that all pairs were rated as unrelated by all raters.

The experiment was created with E-Prime 3.0 (Psychology Software Tools) and made available for download by the subjects with E Prime Go 1.0. In the first experimental session, tasks 1–3 were completed. The encoding phase of each task included 18 word pairs and 18 picture pairs. In the retrieval phase, eight old pairs, which were in the same combination as in the encoding phase, eight recombined pairs consisting of new combinations of words encountered in the encoding phase, and eight entirely new pairs, which had not been studied, were presented for each stimulus type (word and picture pairs).

The second experimental session began with a surprise recognition test including one old, one recombined, and one new pair from each stimulus pair type and from each encoding task of the first session, resulting in 18 pairs. None of these stimulus pairs had been tested within the first session. This delayed recognition test was followed by four additional memory tasks in which 16 word pairs (tasks 5 and 7) or 16 picture pairs (tasks 6 and 8) were learned. In each subsequent recognition test, 8 old, 8 recombined, and 8 new word or picture pairs were presented respectively.

Presentation order of the stimuli in the encoding and the recognition phases of each task was random and different for each participant. All word or picture pairs were presented on the left and right sides of the center of the screen words in black Arial font, size 36, and pictures in gray scale, against a gray background. The display was adjusted to fit the subject’s screen.

### Procedure

All subjects were informed in writing and via phone about the procedure and gave their written consent to participate in the study by e-mail. After making an appointment by e-mail, the elderly subjects were contacted by telephone to complete the Telephone Interview of Cognitive Status (Brandt et al., 1988). Immediately afterward, subjects were sent a link to an online questionnaire, which served to collect demographic and health-related information, as well as to assess several individual difference variables via standardized questionnaires. This included the German versions of the State-Trait-Anxiety Inventory (STAI; Speilberger and Vagg, 1984), the SCD-Questionnaire (SCD-Q; Gifford et al., 2015) including an added question (“Do you experience a significant decrease in your cognitive abilities beyond a normal age-related decline?”, with answer options “I do and I am worried about it,” “I do but I am not worried about it” and “No”), the short version of the NEO-Five Factory Inventory (NEO-FFI30; Körner et al., 2008) the Beck-Depression Inventory V (BDI-V; Schmitt and Maes, 2000), the University of California at Los Angeles Loneliness Scale (UCLA; Döring and Bortz, 1993), three questions regarding the subjective feeling of social isolation before and during the first lockdown due to the Corona-pandemic preceding this study (Shankar et al., 2011), as well as currently (at the end of the second lockdown), the Trier Inventory for Chronic Stress (TICS; Dietzen and Nater, 2006) and the Positive and Negative Schedule (PANAS; Watson et al., 1988). A summary table of these questionnaire measures is presented in Supplementary Material 1. Note that since more than half of the subjects failed to complete the PANAS at some time point, the PANAS was not analyzed.

To complete the first experimental session, the subjects received an e-mail with a link to download a file that contained the first three tasks and that could be run on the participants’ personal computer. In addition, the email included the PANAS questionnaire and a debriefing which strategy they had chosen for task 3 and how helpful it has been. Participants were asked to fill out the PANAS electronically once before and once after completing all experimental tasks, and the strategy questionnaire after completing task 3.

The second experimental session was completed 24 (±2) hours after the first session. This was insured by giving detailed instructions to the participants, sending a separate email with the task file for the second session and additional control of the time stamp of the Eprime file. The second experimental session included the delayed recognition tests for stimuli from the first session, as well as tasks 4–7. The PANAS was filled out only before the beginning of the tasks. In case of technical questions, participants were encouraged to contact the research team by phone. A team member was available for assistance during all participant sessions.

The young subjects were sent the procedures with all associated links in a single email. The time log of the e-prime file was checked to ensure that the 24 h interval with a range of 2 h ± was respected.

Before each session, subjects were informed that they should perform the tasks in a quiet environment and that possible sources of interference should be eliminated.

### Memory tasks

Subjects were told that they were taking part in a study examining subjective well-being and personal preferences. No mention was made that any memory tests were to be completed at any point.

**FIGURE 1:**
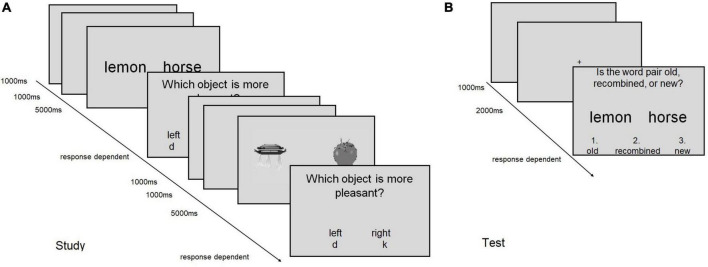
Schematic display of the trial structure in **(A)** the encoding (study) phase, and **(B)** the recognition (test) phase of tasks 1 and 2. **(A)** Two encoding trials are shown, which each consist of the presentation of the stimulus display (5,000 ms), the response prompt (response dependent) and a blank screen (1,000 ms). Between two trials, a fixation cross is shown (1,000 ms). The encoding trial structure applies to all tasks except for task 3, in which no rating screen was shown. **(B)** A recognition trial is shown, in which the correct response would be “old.” The same procedure applies to Task 4–7.

The trial structure was the same for all tasks and is illustrated in Figure 1.

During encoding, a fixation cross of 1,000 ms was followed by the word or picture pair for 5,000 ms. Subsequently, a response prompt was displayed and subjects were asked to respond according to the instructed encoding task (see below). The response terminated the trial and a blank screen appeared for 1000 ms. Self-paced breaks followed each set of 12 or 8 trials for task 1–3 and task 4–7, respectively. The task instructions during encoding were as follows:

#### Encoding task 1

Subjects were asked to rate whether they found the left (key d) or right (key k) word or picture of a pair more pleasant. Subjects were not informed that a memory test would follow; encoding was hence incidental.

#### Encoding task 2

The task instructions were the same as in task 1. However, the following recognition test was explicitly announced before encoding.

#### Encoding task 3

Subjects were asked to memorize each item and each pair for a subsequent recognition test in any manner they chose. This encoding task differed from all other tasks in that no rating screen was displayed after the presentation of the stimulus pairs.

##### Encoding task 4

The subjects were presented with a sentence containing two gaps. They were instructed to mentally complete the sentence with the two words (e.g., presentation of the words dagger – roof “The ___ is smaller than the ___.”) They were asked to rate on a four-point scale [1 (“very well”); 2 (“rather well; 3 = rather poorly”); 4 (“very poorly”)] how well they could complete the sentence.

#### Encoding task 5

Subjects were given a sentence with two gaps. This sentence was intended to help subjects imagine the two pictures in an interaction (e.g., presentation of the picture of a cow and the planet Saturn with the sentence “The ___ lives on the ___.”) They were asked to rate on a four-point scale how well they could imagine the interaction of the objects (see task 4).

#### Encoding task 6

The subjects were instructed to generate their own sentence using the two words and to rate how well they succeeded (see task 4).

#### Encoding task 7

The subjects were asked to generate any interactive image themselves and to rate how well they could imagine an interaction between the objects (see task 4).

Each encoding phase was followed, after a 1-min break, by a recognition test, which exhibited the same structure in all cases. After a fixation cross (2000 ms), the word/picture pair was presented and subjects were asked to indicate whether the word pair was old (key 1), recombined (key 2), or new (key 3). After the answer was given, an ITI (1000 ms) followed (Figure 1).

In between completion of the recognition test and the next task, there was a break for 1 min.

### Data analysis

The proportion of “old,” “new,” and “recombined” responses for each trial type (old, recombined, new) were calculated separately for each task and stimulus material (words vs. pictures, tasks 1–3). Furthermore, PR scores were calculated for item and associative memory separately. For item memory, “old” or “recombined” responses to old or recombined pairs were included as hits. “Old” or “recombined” responses to new pairs counted as false alarms. For the associative memory score, “old” responses to old pairs were counted as hits and “old” responses to recombined pairs were considered as false alarms. PR scores for item and associative memory, separately for pictures and words, were calculated by subtracting the respective false alarm rates from the hit rates (see Kamp and Zimmer, 2015).

Furthermore, to examine the self-reported success of strategy use for tasks 4–7, we analyzed the study ratings. The proportion of the judgments 1 (“very well”) and 2 (“rather well”) were taken as an index of successful application of the strategy for tasks 4–7. Secondly, for task 3 we analyzed the strategy query. Following Dunlosky and Hertzog (1998), the participants’ self-reported strategies were categorized into “imagery,” “repetition,” “sentence generation,” “other,” and “none.” Two independent raters applied this categorization, and any disagreement was dissolved by discussion. Participants were asked to rate the effectiveness of their self-selected strategy on a scale of 1–5 (1 = “very well,” 2 = “good,” 3 = “satisfactory,” 4 = “sufficient,” 5 = “poor”) and the frequencies of each rating were determined.

### Statistical analysis

Data were analyzed using SPSS 27 software. We conducted mixed factors ANOVAs, including age as a between-subjects variable, memory type (item vs. associative) as within-subject variable, and additional within-subject variables according to the hypotheses, as specified in the results section. Of primary interest are interactions of age and memory type, reflecting an age-related associative memory deficit, with additional variables of interest, suggesting that the age-related associative deficit is modulated by these variables. However, other significant main and interaction effects are also reported, because they may provide additional insights, for example into the manner in which the memory tasks were generally solved. To follow up on significant interactions, lower level ANOVAs and planned *t*-tests were calculated. We reported Greenhouse–Geisser corrected degrees of freedom and *p*-values and specify partial eta square (η^2^_p_) as a measure of effect size.

To test whether the age groups differed from each other in the frequency with which specific strategies were self-selected (task 3), chi-square tests were calculated for each of the five categories. We report phi (ϕ) as a measure of effect size.

## Results

### Modulation of the age-related associative deficit by task and stimulus material

**FIGURE 2:**
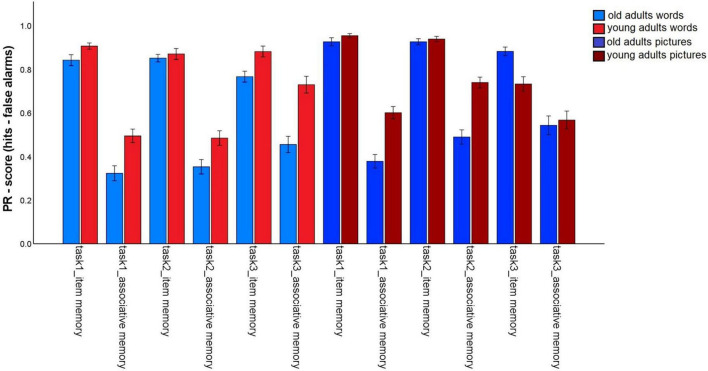
Pr-Scores of item memory and associative memory for words and pictures, separated for younger and older subjects and for the three different tasks in the first session. Task 1: Incidental pleasantness judgment task, task 2: intentional pleasantness judgment task, task 3: free strategy selection. Error bars represents the standard error of the mean.

The first analysis tested whether incidental (task 1) vs. intentional (tasks 2 and 3) encoding, and the provision of a specific task (preference rating: tasks 1 and 2) vs. self-generated encoding strategies (task 3), as well as the nature of the material affects the magnitude of the age-related associative deficit (Figure 2). To do so, we conducted a 2 (memory type: item vs. associative) × 2 (material: words vs. pictures) × 3 (task: 1 vs. 2 vs. 3) × 2 (age group: old vs. young) mixed factors ANOVA. A significant main effect for age group, *F*(1,147) = 16.81, *p* < 0.001, η^2^_p_ = 0.10, and a main effect for memory type, *F*(1,147) = 1013.34, *p* < 0.001, η^2^_p_ = 0.87, were qualified by a significant age group × memory type interaction, *F*(1,147) = 52,69, *p* < 0.001, η^2^_p_ = 0.27. This interaction shows that the typical age-related associative memory deficit was replicated: Older adults showed stronger reductions in associative- than in item memory performance.

**FIGURE 3:**
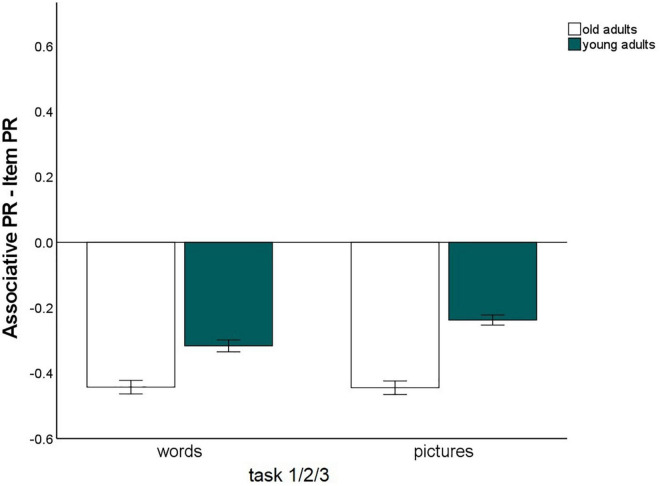
Associative recognition PR, relative to item recognition PR, averaged over tasks 1, 2, and 3 (Difference between associative memory PR and item memory PR). Error bars represent the standard of the error mean.

The main effect for material, *F*(1,147) = 56.25, *p* < 0.001, η^2^_p_ = 0.28, and the material × age group, *F*(1,147) = 16.3, *p* < 0.001, η^2^_p_ = 0.10, and memory type × material, *F*(1,147) = 6.03, *p* = 0.015, η^2^_p_ = 0.04, interactions reached significance and were qualified by a triple memory type × age group × material interaction, *F*(1,147) = 5.59, *p* = 0.019, η^2^_p_ = 0.04. Hence, the magnitude of the associative deficit was modulated by stimulus type. To disentangle this pattern, we calculated the mean item and associative Pr scores across the three tasks, separately for pictures and words, and calculated the difference between item- and associative Pr, thus reflecting associative memory performance relative to item memory performance. A significant age difference was found in this measure between the age groups for pictures, *t*(147) = –7.25, *p* < 0.001, and for words, *t*(147) = –4.57, *p* < 0.001, indicating that there was a disproportionate age-related associative deficit for both kinds of material. However, the age difference was larger for pictures than for words (Figure 3). No other interactions involving the factors age group and memory type reached significance, suggesting that in this analysis, the age-related associative deficit was robustly modulated only by stimulus material.

The ANOVA also revealed a triple interaction task × material × group, *F*(1.85,271.15) = 29.26, *p* < 0.001, η^2^_p_ = 0.17. To better understand this interaction, we calculated the mean performance across item and associative memory, separately for each task and material type. Then, a 3 (task) × 2 (age group) ANOVA was calculated separately for words and pictures.

For words, significant main effects were found for task, *F*(1.78,261.4) = 7,57, *p* = 0.001, η^2^_p_ = 0.05, and age group, *F*(1,147) = 24.62, *p* < 0.001, η^2^_p_ = 0.14. The interaction was also significant, *F*(1.78,261.4) = 4.57, *p* = 0.014, η^2^_p_ = 0.03. For older subjects, overall memory performance did not differ between tasks (task 1: *M* = 0.58; task 2: *M* = 0.60; task 3: *M* = 0.61; all *p*-values > 0.05). The memory performance of younger adults did not differ between tasks 1 (*M* = 0.7) and 2 (*M* = 0.68; *p* = 0.32), but significantly increased for task 3 (*M* = 0.8; both *p*-values < 0.005).

For pictures, the ANOVA revealed a main effect for task, *F*(1.55,227,91) = 10,53, *p* < 0.001, η^2^_p_ = 0.07 and age group, *F*(1,147) = 7,36, *p* = 0.007, η^2^_p_ = 0.05, and an interaction, *F*(1.55,227,91) = 14,65, *p* < 0.001, η^2^_p_ = 0.09. For older adults, there was little evidence for difference in overall task performance [while task 1: *M* = 0.65, differed from task 2: *M* = 0.71, *t*(74) = –2.48, *p* = 0.015, the differences among all other task pairs was not significant; task 3: *M* = 0.71, both *p*-values > 0.057]. For the young adults, overall performance decreased from tasks 1 (*M* = 0.78) and 2 (*M* = 0.84) to task 3 (*M* = 0.65; all *p*-values < 0.005). Taken together, young adults’ performance increased for words, but decreased for pictures, when participants chose their own encoding strategies, while this pattern was not found for older adults.

Finally, a memory type × task interaction, *F*(1.98,291.16) = 52.03, *p* < 0.001, η^2^_p_ = 0.26, and a material × task interaction, *F*(1.85,271.15) = 27.11, *p* < 0.001, η^2^_p_ = 0.16, were qualified by a three-way memory type × material × task interaction, *F*(1.87,274.89) = 8.1, *p* = 0.001, η^2^_p_ = 0.05. The three-way interaction suggests that item and associative memory were differentially affected by the task manipulation, and that the nature of this relationship depended on the stimulus material.

For words, item memory was lower in task 3, compared to tasks 1 and 2 (both *p*-values < 0.05), whereas tasks 1 and 2 did not differ, *t*(148) = 0.764, *p* = 0.446. By contrast, associative memory was higher in task 3 than in tasks 1 and 2 (both *p*-values < 0.001), and did not differ between tasks 1 and 2, *t*(148) = –0.38, *p* = 0.705. Hence, allowing participants to generate their own pair encoding strategies specifically enhanced associative memory for words.

For pictures, item memory showed the same pattern as for words, with better memory in tasks 1 and 2 than in task 3 (both *p*-values < 0.001), but no difference between tasks 1 and 2, *t*(148) = 0.59, *p* = 0.556. By contrast, associative memory improved for task 2, compared to task 1, *t*(148) = –5.56, *p* < 0.001. Associative memory for task 3 was in between, but did not significantly differ from either task 1, *t*(148) = –1.93, *p* = 0.056, or 2, *t*(148) = 1.81, *p* = 0.073. Hence, associative memory for pictures benefited from intentional (vs. incidental) encoding in general.

### Influence of the level of guidance

**FIGURE 4:**
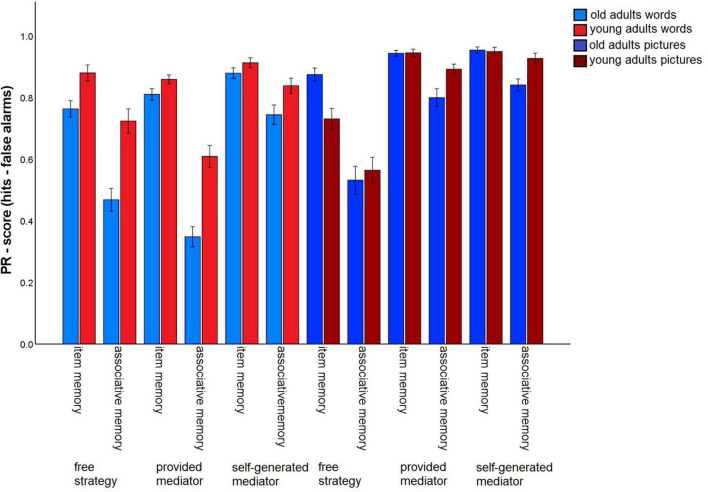
Pr-Scores of item memory and associative memory for words and pictures, separated for young and older subjects and for the three different levels of guidance. Task 3 is the free strategy selection task of the first session. Task 4: Sentence generation task with provided mediators, task 6: sentence generation task with self-generated mediators, task 5: interactive imagery task with provided mediators, task 7: interactive imagery task with self-generated mediators. Error bars represents the standard of the error mean.

To assess whether the instruction of strategies with different levels of guidance (no guidance/task 3 vs. some guidance through strategy instruction, but subjects have to generate their own mediators/tasks 6 and 7 vs. strong guidance by strategy instruction including the provision of specific mediators/tasks 4 and 5) affects the associative deficit and to what extent the material has an impact on this relationship, we conducted a 2 (memory type: item vs. associative) × 2 (material: words vs. pictures) × 3 (level of guidance: free strategy selection vs. strategy instruction with mediators provided vs. strategy instruction with mediators self-generated) × 2 (age group: old vs. young) ANOVA (Figure 4). All main effects were significant [memory type: *F*(1,140) = 317.35, *p* < 0.001, η^2^_p_ = 0.69; material: *F*(1,140) = 118.73 *p* < 0.001, η^2^_p_ = 0.46, level of guidance: *F*(1.37,191.24) = 75.83, *p* < 0.001 η^2^_p_ = 0.35; age group: *F*(1,140) = 10.73, *p* = 0.001, η^2^_p_ = 0.07].

The memory type × age group interaction, *F*(1,140) = 38.26, *p* < 0.001, η^2^_p_ = 0.22, reflecting the age-related associative deficit, was qualified by a three-way interaction of level of guidance × memory type × age group, *F*(1.62,226.82) = 3.52, *p* = 0.041, η^2^_p_ = 0.03. Level of task guidance therefore influenced the magnitude of the age-related associative deficit.

**FIGURE 5:**
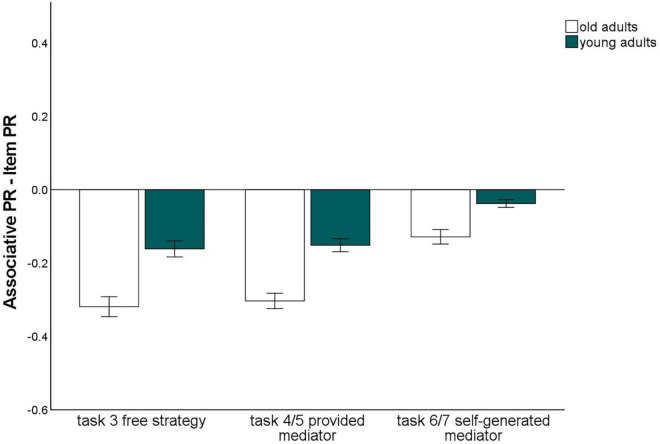
Associative recognition PR, relative to item recognition PR, averaged over material types within each level of guidance. Error bars represent the standard of the error mean.

To resolve this interaction, we calculated the difference between item and associative memory Pr, averaging over material type, and compared the three levels of guidance with *t*-tests for dependent samples (Figure 5). This measure differed between younger and older adults for every level of guidance (*p*-values < 0.005), indicating that an associative deficit was always observed. However, the magnitude of the associative deficit differed dependent on the level of guidance: The age difference was largest for the free strategy selection task (*M* = –0.17), only slightly smaller with the strategy instruction with mediators provided (*M* = –0.14), and was lowest for the strategy instruction with self-generated mediators (*M* = –0.07; Figure 5). No other interactions involving both the factors age group and memory type were observed. This pattern suggests that older adults’ associative memory generally benefited from strategy instruction when participants were allowed to generate their own mediators. However, when mediators were provided, the age-related associative deficit was comparable to a condition in which no strategy instructions were given.

The ANOVA also revealed a significant material x age group interaction, *F*(1,140) = 53.11, *p* < 0.001, η^2^_p_ = 0.28, qualified by a three-way level of guidance × material × age group interaction, *F*(1.79, 250.17) = 13.50, *p* < 0.001, η^2^_p_ = 0.09. To gain a more detailed understanding of this pattern, the mean value across item and associative Pr was calculated for every level of guidance and material type. Then, a 3 (level of guidance) × 2 (age group) ANOVA was calculated separately for words and pictures.

For the words, significant main effects were found for level of guidance, *F*(1.72,241.4) = 53.83, *p* < 0.001, η^2^_p_ = 0.28, and age group, *F*(1,140) = 29.41, *p* < 0.001, η^2^_p_ = 0.17. The interaction was also significant, *F*(1.72,241.4) = 5.84, *p* = 0.005, η^2^_p_ = 0.04. For older subjects, overall memory performance was better for the strategy instruction without provided mediators (*M* = 0.81), compared to both other conditions (all *p*-values < 0.001), with no difference between the latter two [free strategy selection: *M* = 0.62; provided mediators: *M* = 0.58, *t*(69) = 1.24, *p* = 0.22]. Similarly, younger adults also showed the best performance for the strategy with self-generated mediators (*M* = 0.88), followed by the free selection strategy (*M* = 0.80) and the strategy with provided mediators (*M* = 0.73; all *p*-values < 0.05).

For pictures, the ANOVA revealed a main effect for level of guidance, *F*(1.28,179.18) = 102.15, *p* < 0.001, η^2^_p_ = 0.42, and level of guidance x age group interaction, *F*(1.28,179.18) = 4.74.83, *p* = 0.022, η^2^_p_ = 0.03. For older and younger subjects the same general pattern was observed: Performance in the free strategy selection condition (old: *M* = 0.70; young: *M* = 0.65) was lower than for the strategy instruction with mediators provided (old: *M* = 0.87; young: *M* = 0.92) and the strategy instruction with self-generated mediators (old: *M* = 0.90; young: *M* = 0.94) (all *p*-values < 0.001), with no difference between the latter two (both *p*-values > 0.092). Thus, both age groups appeared to benefit from both strategy instruction conditions for pictures, but the magnitude of the overall performance increase was larger for young than for older adults. It is important to note that this pattern concerns overall memory performance, and not the relative associative memory deficit in older adults.

In the overall ANOVA, furthermore, interactions between memory type × material, *F*(1,140) = 30.02, *p* < 0.001, η^2^_p_ = 0.18, memory type x level of guidance, *F*(1.28,226.82) = 49.99, *p* < 0.001, η^2^_p_ = 0.26, and material × level of guidance, *F*(1.79,250.17) = 83.03, *p* < 0.001, η^2^_p_ = 0.37, were qualified by a significant triple interaction between memory type × material × level of guidance, *F*(1.99,278.83) = 32.61, *p* < 0.001, η^2^_p_ = 0.19. To resolve the interaction, 3 (level of guidance) × 2 (memory type) ANOVAs were calculated separately for words and pictures.

For words, besides main effects for level of guidance, *F*(1.76,248.66) = 51.63, *p* < 0.001, η^2^_p_ = 0.27, and memory type, *F*(1,141) = 235.23, *p* < 0.001, η^2^_p_ = 0.63, the interaction of both factors reached significance, *F*(1.9,267.5) = 37.12, *p* < 0.001, η^2^_p_ = 0.21. Item memory performance did not differ between free strategy selection (*M* = 0.82) and strategy instruction with mediators provided (*M* = 0.84), *t*(141) = –0.64. *p* = 0.522. However, item memory was better for the strategy instruction with self-generated mediators (*M* = 0.90), compared to both other levels of guidance (both *p*-values < 0.001). Associative memory also benefited most from strategy instruction with self-generated mediators (*M* = 0.79), but performance decreased to free strategy selection (*M* = 0.60) and to strategy instruction with provided mediators (*M* = 0.48) (all *p*-values < 0.001).

For pictures, both main effects level of guidance: *F*(1.27,179.16) = 100.13, *p* < 0.001, η^2^_p_ = 0.42, memory type, *F*(1,141) = 107.86, *p* < 0.001, η^2^_p_ = 0.43, and the interaction, *F*(1.73,243.66) = 43.13, *p* < 0.001, η^2^_p_ = 0.23, were significant. For item memory, both strategy instruction conditions (provided mediators: *M* = 0.95; self-generated mediators: *M* = 0.95) produced better memory than the free strategy selection (*M* = 0.80) (both *p*-values < 0.001), while the former two did not differ, *t*(141) = –0.9, *p* = 0.37. For associative memory, performance was best for the instructions with self-generated mediators (*M* = 0.89), intermediate for the provided mediators (*M* = 0.85) and lowest for the free strategy selection (*M* = 0.55, *SD* = 0.37). All comparisons were significant (all *p*-values < 0.05).

### Immediate versus 24 h-delayed retrieval

**FIGURE 6:**
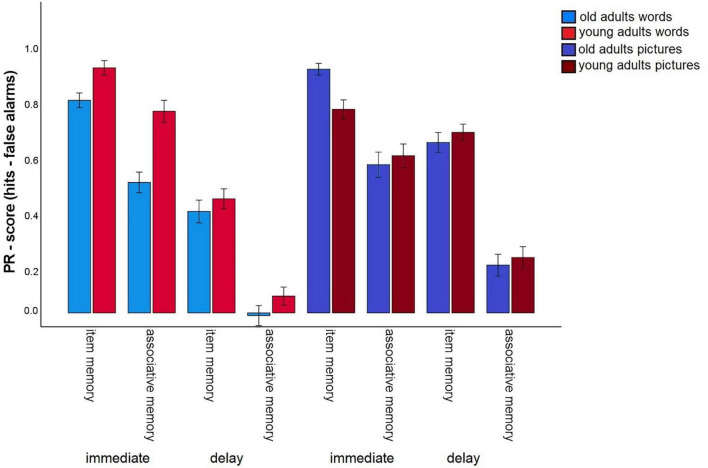
Pr-Scores of item memory and associative memory for immediate (average task 1–3) and delayed retrieval, separated for younger and older subjects, and for words and pictures. Error bars represent the standard error of the mean.

**FIGURE 7:**
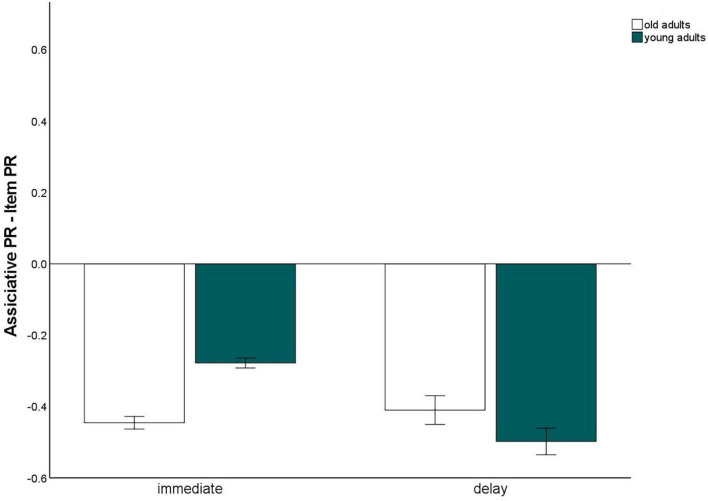
Associative recognition PR, relative to item recognition PR, averaged over material types within immediate and delayed retrieval. Error bars represents the standard of the error mean.

To gain measures of immediate recognition performance, we collapsed recognition performance for the first experimental session across all tasks, separately for item and associative memory and for pictures and words. The same was done for the delayed recognition test. We calculated a 2 (material type: words vs. pictures) × 2 (test delay: immediate vs. 24 h delayed) × 2 (memory type: item vs. associative contrast) × 2 (age group: old vs. young) mixed factors ANOVA to test whether the test delay (and material type) affects the age-related associative deficit (Figure 6). This ANOVA revealed significant main effects for material, *F*(1,140) = 114.65, *p* < 0.001, η^2^_p_ = 0.45, delay *F*(1,140) = 787.04, *p* < 0.001, η^2^_p_ = 0.85, memory type, *F*(1,140) = 823.78, *p* < 0.001, η^2^_p_ = 0.86, and age group, *F*(1,140) = 9.4, *p* = 0.003, η^2^_p_ = 0.06. The significant interaction between memory type × age group, *F*(1,140) = 10.14, *p* = 0.002, η^2^_p_ = 0.07, reflecting the age-related associative deficit, was qualified by a three-way interaction for delay × memory type × age group, *F*(1,140) = 8.05, *p* = 0.005, η^2^_p_ = 0.05. This interaction revealed that the associative deficit was evident with immediate tests, since the difference between item and associative Pr, averaged across pictures and words, differed between the age groups, *t*(147) = –7.44, *p* < 0.001, but not with delayed tests, *t*(147) = 1.6, *p* = 0.11 (Figure 7). Importantly, this pattern was not due to floor effects in associative memory performance (Figure 2), since the average associative Pr significantly differed from 0 for both age groups [old: *t*(69) = 3.17, *p* = 0.002; young: *t*(71) = 5.34, *p* < 0.001]. No other interactions involving both age group and memory type were significant.

The ANOVA also revealed a material x delay interaction, *F*(1,140) = 33.36, *p* < 0.001, η^2^_p_ = 0.19, qualified by a three-way interaction material × delay × memory type, *F*(1,140) = 5.68, *p* = 0.019, η^2^_p_ = 0.04. Therefore, the timing of the recognition task appeared to affect item and associative memory in different ways and the nature of this relationship depended on the stimulus material. To test this, separate 2 (delay: immediate vs. 24 h delay) × 2 (memory type: item vs. associative memory) ANOVAs were calculated for words and pictures.

For words, a main effect for delay, *F*(1,141) = 670.72, *p* < 0.001, η^2^_p_ = 0.83, and a main effect for memory type, *F*(1,141) = 348.41, *p* < 0.001, η^2^_p_ = 0.71, but no interaction between these factors *F*(1,141) = 0.20, *p* = 0.65, η^2^_p_ = 0.001, were found. Hence, item and associative memory for words declined about equally when memory was tested with a 24 h delay.

For pictures, in addition to the significant main effects (both *p*-values < 0.001), a significant interaction between delay × memory type was found, *F*(1,141) = 8.35, *p* = 0.004, η^2^_p_ = 0.06. This interaction shows that item memory for pictures declined at a lower rate due to the delay (immediate: *M* = 0.89; 24 h delay: *M* = 0.63) than associative memory (immediate: *M* = 0.55; 24 h delay: *M* = 0.19).

### Analysis of study ratings

**FIGURE 8:**
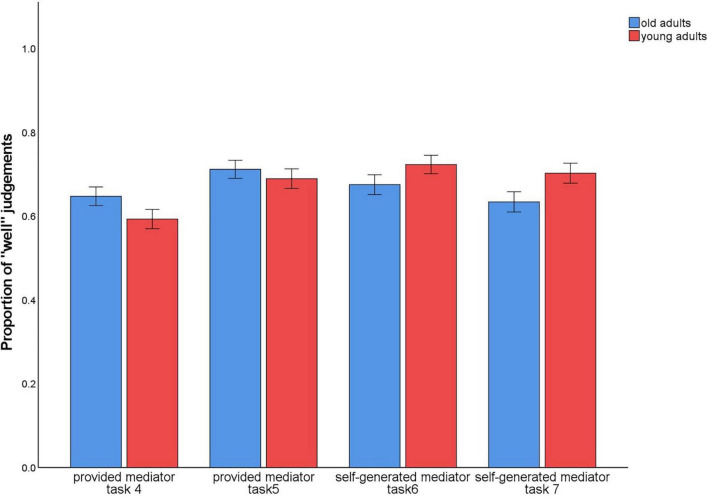
Proportion of trials in which participants provided the ratings 1 (“very well”) or 2 (“rather well”) to the question of how well they could complete the sentence or imagine the interaction during encoding. Provided mediator: a sentence describing an interaction of the two words (task 4) or pictures (task 5) was given. Self-generated mediator: the sentence describing the interaction of the two words (task 6) or pictures (task 7) was to be generated by the participants themselves. After each presentation of the word or picture pairs and the given or self-generated sentence, a rating was given of how well participants could envision the sentence or imagine the interaction: 1 (“very well”); 2 (“rather well”); 3 (“rather poorly”); 4 (“very poorly”).

In tasks 4–7, subjects evaluated in each trial how well they could envision the sentence with the two words or imagine the interaction of the objects. To check whether these ratings differed between the conditions with the provided mediators vs. the self-generated mediators, we calculated the proportion of trials in which participants provided the rating 1 (“very well”) or 2 (“rather well”). On these values, a 2 (level of guidance: provided/4 and 5 vs. self-generated/6 and 7) × 2 (material type: words vs. pictures) × 2 (age group: old vs. young) ANOVA was calculated. There was a main effect for material type, *F*(1,140) = 4.45, *p* = 0.037, η^2^_p_ = 0.03, and an interaction between level of guidance × material type, *F*(1,140) = 29.07, *p* < 0.001, η^2^_p_ = 0.17 (Figure 8).

For words, the provided mediators (*M* = 0.62) were less likely to be rated as easy to envision than the self-generated mediators (*M* = 0.69), *t*(141) = –4.44, *p* < 0.001. The opposite pattern was observed for pictures (provided: *M* = 0.70; self-generated: *M* = 0.67), *t*(141) = 2.24, *p* = 0.027.

Importantly, there was no main or interaction effect involving the factor age group, (both *p*-values < 0.005). Hence, according to the ratings provided during encoding, the strategy instructions did not differentially affect how well the strategies were applied in young vs. older adults.

### Report of the self-generated strategies

In the debriefing completed after the task in which participants used their own strategies, seven older participants reported using two strategies; in these cases, both strategies were included in the observed category frequencies. Younger adults were more likely to report using sentence generation compared to older adults, χ^2^ (1, *N* = 111) = 12.74, *p* = 0.001, ϕ = 0.34. The older subjects more often reported that they did not use any strategy, χ^2^ (1, *N* = 111) = 8.81, *p* = 0.003, ϕ = 0.28. In the remainder of the categories, there was no age group difference (all *p*-values > 0.05) (see Supplementary Figure 1). In addition, the age groups differed in their rating of how well their self-selected strategy helped them recall the pairs later, χ^2^ (4, *N* = 111) = 19.27, *p* = 0.001, ϕ = 0.42. Older subjects were more likely to give a “satisfactory” rating, while younger subjects were more likely to rate their strategy as “good.”

## Discussion

In a within-subjects design, we investigated the extent to, and the manner in, which the age-related associative deficit in episodic memory is modulated by stimulus presentation format (words vs. pictures), incidental vs. intentional encoding, test delay and strategy instructions with different levels of guidance, placing different demands on self-initiated strategic processing. A robust associative deficit was found for all encoding conditions. We found no evidence for a modulation of the age-related associative deficit by encoding intentionality. However, the nature of the stimulus material and the test delay significantly modulated the magnitude of the age-related associative deficit. Furthermore, item and associative memory of both age groups benefited from the provision of effective strategies, and the age-related associative deficit was reduced in the sentence generation and interactive imagery tasks when participants were allowed to generate their own mediators. The results of the present study contribute to a better understanding of the modulating factors for the associative memory deficit in aging, as will be discussed in detail in the remainder of this article.

### Incidental versus intentional encoding

The age-related associative memory deficit was about equally pronounced in incidental and intentional learning tasks; we found no evidence for an effect of encoding intentionality on the associative deficit. This is not consistent with meta-analytic findings that older adults’ associative deficit is smaller in incidental encoding (Spencer and Raz, 1995; Old and Naveh-Benjamin, 2008).

We kept the encoding task instruction (comparative pleasantness judgment) constant between incidental and intentional encoding in tasks 1 and 2. Keeping the task instructions equal presumably allows for a comparable processing depth, such that the only difference between the tasks is whether a subsequent memory test is expected. No evidence for an effect of encoding intentionality was found in this comparison. In prior studies testing the effect of intentional vs. incidental encoding on the age-related associative deficit, different learning instructions were given, such as “learn the names and faces for a later memory test” in the intentional task, and different cover stories in the incidental tasks, such as “does the name match the face” or describing a salient feature of the faces or naming the first letter of the name (Troyer et al., 2006; Naveh-Benjamin et al., 2009). In our study, a more similar comparison may be between the incidental encoding task (task 1) and the intentional task (task 2) in which the instruction was only to learn the items (task 3). In this comparison, however, we also found no evidence that the magnitude of the associative deficit was modulated by encoding intentionality.

Notably, our instruction for incidental encoding required an affective comparison between the items of a pair. This task presumably leads to relatively deep processing, even when encoding is incidental (Craik and Tulving, 1975). Perhaps in incidental tasks that engage shallower, and more item-specific processing, encoding intentionality would more strongly affect the age-related associative deficit, a possibility that has to be examined in the future.

It is also interesting to note that young adults showed strongly improved associative memory for word pairs when they generated their own strategies, but showed a reduction in both item and associative memory performance for pictures in this condition (Figure 2). This pattern was not found for older adults, which could be a result of the stronger inclination of young adults to apply sentence generation strategies, which may not be as effective with pictures. It thus would be interesting to examine whether the present results regarding encoding intentionality would replicate if stimulus materials were homogeneous within a task, rather than including both word and picture pairs in a random sequence.

A caveat of the present study is that the order of task completion was always the same, such that the intentional tasks always followed the incidental task. Hence, we cannot rule out that practice or fatigue effects could modify the manner in which encoding intentionality influences the age-related associative deficit. However, it is worth pointing out that item and associative memory were differentially affected by the task manipulation: While item memory declined from incidental encoding to intentional encoding with self-chosen strategies, associative memory (at least for words) generally improved in the same comparison, a finding that was common to both age groups (Figure 2). Relatively simple explanations of general practice or fatigue effects are hence unlikely to account for our findings, although more complex task order effects cannot be ruled out.

Taken together, the present data do not support the view that the age-related associative deficit is generally smaller in incidental than in intentional encoding conditions. A dependency of the effect on encoding intentionality on the associative memory deficit on the instructed incidental encoding task remains to be investigated in future studies.

### Role of strategies

An interesting finding emerges from the debriefing of Task 3. In this task, participants had the opportunity to self-generate their own strategies, and notable differences between age groups emerged. Young adults were more likely to report using sentence generation, while older adults were more likely to report an undifferentiated encoding strategy, which they also rated as less helpful compared to the younger adults. It should be noted, however, that we used retrospective reports of strategy use and that other studies using item-by-item reports have not found age differences in reports of strategy use (Dunlosky and Hertzog, 1998).

Importantly, these results suggest that older adults are less likely to use effective encoding strategies spontaneously as do young adults, underscoring the importance of strategic factors in the age-related deficit in associative memory (Shing et al., 2010; Craik and Rose, 2012). Thus, the present results show that the instruction to self-generate a sentence or to use interactive imagery during pair encoding leads to improved memory performance especially for associative information in both age groups, and that the age-related associative memory deficit can be alleviated with strategy instruction (task 3 vs. tasks 6/7).

In the present study, we focused on encoding strategies, but it is important to note that strategic processes during retrieval could also play a role (Cohn et al., 2008). In our study, when participants could generate their own strategies, older individuals rated them as less helpful. Furthermore, older subjects reported using multiple strategies more often than younger individuals, which may have led to insufficient orientation to the chosen strategy during retrieval. Thus, associative memory performance, especially in older adults, may be further enhanced by strategy instruction at retrieval, for example if reference was made to the encoding instruction prior to retrieval (Naveh-Benjamin et al., 2007).

Enhancing the level of guidance by specifying mediators for the provided strategies did not in all cases lead to an improvement in memory and also did not lead to a further alleviation of the associative deficit. Thus, while for interactive imagery, provision of mediators led to an about equal overall memory performance to merely instructing the strategy (although this did not further alleviate the relative associative deficit), for sentence generation, providing the mediators impaired associative memory. All in all, a higher level of guidance generally did not lead to an alleviation of the associative deficit in the present study.

Importantly, both age groups reported that they were not easily able to envision the sentences provided as mediators while the provided interactive images were rated as relatively easy to imagine. Hence, it appears that in order to support older adults’ associative memory, the given mediators must be chosen in such a way that the older adults can apply them easily. Otherwise, a conflict with prior semantic knowledge may interfere with the formation of an association (Naveh-Benjamin, 2000; Kamp et al., 2018). The younger adults, by contrast, perhaps had an easier time forming an association despite the poor applicability of the sentence mediators, suggesting that their encoding processes were flexible enough to cope with the difficulty in applying the mediator. Notably, a similar finding was reported by Bastin et al. (2013; experiment 1).

Again, we cannot rule out practice effects, because the tasks were always completed in the same order. This design was chosen because we reasoned that it would be difficult to first self-generate mediators and only afterward complete the same task with provided mediators, as the latter task may be perceived as more constraining and obstructive in this order. A strong influence of practice effects on our result pattern, however, appears to be unlikely, as older subjects’ associative memory improved strongly from task 4 to 6 for the sentence task, but not as strongly from task 5 to 7 for interactive imagery. Nevertheless, strategy training effects, dependent on the level of guidance that a strategy provides, remain a fruitful route for ongoing and future research (Lövdén et al., 2012; Brehmer et al., 2016).

It is important to note that prior studies did find that older adults’ associative memory can benefit from encoding or retrieval guidance, for example through manipulations of unitization, the process of creating a unified whole out of the constituents of an association (Graf and Schacter, 1989). However, it appears that older adults benefit from unitization mostly when it is supported by prior knowledge (Ahmad et al., 2014) or is relatively easy to apply (Bastin et al., 2013), but not when the strategy may be in conflict with pre-existing semantic knowledge (Bridger et al., 2017; Kamp et al., 2018).

Taken together, the present study joins others in demonstrating that strategy instruction can help older adults increase recognition of associative information such that it may nearly match the memory performance of younger adults. However, task characteristics must be carefully chosen. Thus, when mediators are provided, they should be easily and plausibly applicable in order for older adults to benefit from their provision.

### Material type

In the present study, the type of material influenced the age-related associative memory deficit. The cleanest comparison between material types was in tasks 1–3, because here, the task instructions were identical for words and pictures. Here, as we hypothesized, the associative deficit was less pronounced for words than for pictures. In contrast, Guez and Lev (2016) reported data suggesting that the associative deficit is independent of content. The reason for this discrepancy between these findings is unclear, but our data do suggest that more research examining the impact of stimulus modality on the age-related associative deficit would be fruitful.

A notable feature of our task (tasks 1–3) was that we presented the pictures and words intermixed in a random sequence. Maybe the unpredictable switch between the material types influenced the processing of the items and their associations. Older adults show increased general switching costs when different task requirements have to be maintained and organized (Kray and Lindenberger, 2000; Wasylyshyn et al., 2011). Hence, for example, if older adults speak the words to themselves during encoding, when a picture pair follows a word pair, they may do the same with pictures. Since older adults need longer search times to name pictures and often make more mistakes than younger adults (Hodgson and Ellis, 1998), this could impair the formation of a successful connection and disrupt relational processing. Hence, future studies should examine the effects of stimulus modality on the associative deficit when picture pairs or word pairs are blocked in separate lists.

Interestingly, the effect of stimulus type on the associative deficit was not significant in task 4–7, so that, if anything, there tended to be a less pronounced associative deficit for the pictures. In these tasks, either only words or pictures were presented, again suggesting that whether stimulus types are intermixed or blocked may influence the impact of stimulus modality on the associative deficit. However, it is difficult to disentangle the influence of the instructed strategy from stimulus modality in task 4–7.

Taken together, our results suggest that the magnitude of the age related associative deficit may depend on the nature of the stimulus material, but this effect may be modulated by task characteristics. Further studies should also examine how other manipulations of stimulus modality may affect the associative memory deficit in aging. For example, differences in the nature and endurance of auditory vs. visual memories have recently received attention (Gloede and Gregg, 2019). It would hence be fruitful to examine whether and how the age-related associative deficit is modulated by auditory vs. visual presentation modalities.

### Test delay

Although the age-related associative deficit was pronounced and robust with immediate testing, in the present results no age-related associative deficit was found after a delay of 24 h. In fact, in the delayed test there were unexpectedly no age differences in either item or associative memory. In principle, a possible reason could be that older people may be generally more motivated in memory experiments because of a personal relevance and hence a deeper encoding could have led to the newly generated memories being more robust to forgetting than in young adults (Greene and Naveh-Benjamin, 2022). However, if this is correct, it would be difficult to explain the superior memory performance of young adults, and the pronounced age-related associative memory deficit, in immediate tests. We hence consider the present finding as preliminary. It is thus important for future studies to replicate and further examine the effect of delayed memory testing on age differences in item and associative memory.

One limitation of our study is that a low number of stimuli were included in the delayed test, preventing a systematic investigation of the influence of incidental and intentional learning and of encoding instructions on the effect of test delay on the age-related associative deficit. Since these factors could offer an explanation for the absence of the age-related associative memory deficit with a 24 h delayed test, future studies should examine these factors in greater detail.

## Limitations and conclusion

In this study, several potential mediators of the age-related associative deficit were examined in a within subject design. A limitation of our study is that the tasks were always completed in the same order. Hence, we cannot rule out order effects including practice and fatigue effects. Especially the provision of specific mediators followed by completion of the same tasks with self-generated mediators (tasks 4 and 5 vs. 6 and 7) could have led to practice effects. Practice effects should hence be more systematically considered in further studies.

Furthermore, another limitation is that the learning phase in tasks 1–3 included both words and pictures in a randomly intermixed sequence, and hence included about twice as many stimuli than tasks 4–7. Follow-up studies could present word and picture pairs in separate learning phases with task instructions otherwise comparable to tasks 1–3.

In conclusion, the present study sheds new light onto the modulators of the age-related associative deficit. Our results do not support the view that the age-related associative deficit is modulated by an intentional versus incidental encoding manipulation. However, the results join others to demonstrate that the appropriate selection of strategy instructions can enhance memory performance in older adults and minimize the age-related deficit. It appears important that the instructed strategies are plausible or consistent with prior semantic knowledge, such that older subjects can use them to their advantage. Furthermore, the type of material is of crucial importance and may lead to age differences depending on the task characteristics. Finally, our results provide some indications that the age-related associative deficit may be attenuated when memory is tested after a delay.

## Data availability statement

The datasets presented in this study can be found in online repositories. The names of the repository/repositories and accession number(s) can be found below: https://osf.io/dt7zj/?view_only=b3a17d39a42f438983301abe0c7932c2.

## Ethics statement

The study involving human participants were reviewed and approved by the Ethics Committee of the University of Trier. The patients/participants provided their written informed consent to participate in this study.

## Author contributions

RE and S-MK contributed to the conception and design of the study. RE coordinated data collection and data entry and performed the statistical analyses and data visualization under the supervision of S-MK. Both authors wrote the manuscript collaboratively and approved the final submission.
