# Supplementary material for: An examination of task factors that influence the associative memory deficit in aging
Source: Front Psychol. 2022 Sep 23;13:991371. doi: 10.3389/fpsyg.2022.991371 (PMC9539925; doi:10.3389/fpsyg.2022.991371)
Supplement: Supplementary file 1 [file Data_Sheet_1.docx]

Supplementary Material

# Supplementary Table 1. Demographic Information and additional questionnaires

Table 1. Sample description: age, sex, education, neuropsychological, psychological test measures of old and young adults; Mean (+/-SD)

|  | ***N*** | ***old adults*** | ***young adults*** |  |
| --- | --- | --- | --- | --- |
| age | 75/74 | 68.17 (6.44) | 25.54 (5.1) |  |
| sex | 75/74 | 37 male / 38 female | 24 male / 50 female |  |
| TICS | 75/-- | 37.47 (3.91) |  |  |
| education (years) | 74/74 | 11.5 (1.99) | 12.4 (1.1) | ** |
| NEO-FFI30: |  |  |  |  |
| neuroticism (N) | 74/74 | 5.43 (4.25) | 9.25 (5.07) | ** |
| extraversion (E) | 74/74 | 14.76 (2.95) | 14.31 (4.3) |  |
| openness to experience (O) | 74/74 | 15.2 (4.42) | 15 (5.56) |  |
| agreeableness (A) | 74/74 | 20 (2.49) | 18.1 (4) | * |
| conscientiousness (C) | 74/74 | 25.26 (2.74) | 18.76 (3.4) | * |
| BDI-V | 74/74 | 14.93 (10.09) | 24.85 (14.62) | ** |
| STAI | 74/74 | 31.3 (7.37) | 39.46 (9.87) | ** |
| SCD-Q | 74/74 | 32.69 (7.56) |  |  |
| SCD *(supplementary question)* | 74/74 | 2.78 (.53) |  |  |
| UCLA | 74/74 | 10.78 (5.59) | 13.26 (7.19) | * |
| Social Isolation Questions T1 | 74/74 | 5.61 (.84) | 5.41 (.55) |  |
| Social Isolation Questions T2 | 74/74 | 5.49 (.88) | 5.32 (.62) |  |
| Social Isolation Questions T3 | 74/74 | 5.63 (.85) | 5.51 (.56) |  |
| TICS *(Trier Inventory of Chronic Stress)* | 74/74 | 12.1 (6.28) | 20.92 (8.69) | ** |

*Note:* TICS =Telephone Interview of Cognitive Status (maximum value: 50; higher values index better cognitive function) (Brandt et al., 1988); NEO-FFI30 = neuroticism, extraversion, openness to experience, agreeableness, conscientiousness (Körner et al., 2008); BDI-V = Beck Depression Inventory-V (Schmitt & Maes, 2000); STAI = German Version of the State and Trait Anxiety Inventory (State Subscale) (Spielberger & Vagg, 1984); SCD-Q = Subjective Cognitive Decline Questionnaire (Range: 22-47) (Gifford et al., 2015); SCD supplementary question: “Do you experience a significant decrease in your cognitive abilities beyond a normal age-related decline?”, with answer options “I do and I am worried about it”, “I do but I am not worried about it” and “No” (Range: 1-3; higher values index a stronger self-perceived cognitive decline for both SCD values); UCLA = University of California at Los Angeles Loneliness Scale (UCLA; Döring & Bortz, 1993),; Social Isolation Questions: three questions regarding the subjective feeling of social isolation before and during the first lockdown due to the Corona-pandemic preceding this study (Shankar et al., 2011), as well as currently (at the end of the second lockdown) TICS = Trier Inventory of Chronic Stress (Dietzen & Nater, 2006)

For all questionnaires: Unless otherwise noted, higher values index a higher degree of the measured construct.

Age group differences: * p < .05. ** p < .001.

Missing data from one older participant who did not complete the online survey of psychological tests.

Education (years): Many older adults mostly reported years of schooling rather than total years of education.

# Supplementary Table 2. PR – scores for item and associative memory

Table 2 Mean (+/- SD) PR-scores for item and associative memory

| ***task*** | ***group*** | ***item memory words*** | ***item memory pictures*** | ***associative memory words*** | ***associative memory pictures*** |
| --- | --- | --- | --- | --- | --- |
| task 1 | old (n = 75) | .84 (.22) | .93 (.16) | .32 (*.30)* | .38 (.27) |
|  | young (n = 74) | .91 (.12) | .95 (.08) | .49 (*SD* = .27) | .60 (.24) |
| task 2 | old (n = 75) | .85 (.15) | .93 (.12) | .35 (.29) | .49 (*.* 28) |
|  | young (n = 74) | .87 (.21) | .94 (.10) | .49 (.29) | .74 (.21) |
| task 3 | old (n = 75) | .77 (.22) | .88 (.17) | .46 (.32) | .54 (.37) |
|  | young (n = 74) | .88 (.22) | .73 (.29) | .73 (.33) | .57 (.36) |
| task delay | old (n = 70) | .36 (.34) | .61 (.30) | .-.01 (.30) | .17 (.33) |
|  | young (n = 72) | .41 (.30) | .65 (.25) | .06 (.28) | .20 (.33) |
| task 4 | old (n = 70) | .81 (.15) |  | .35 (*SD* = .27) |  |
|  | young (n = 72) | .86 (.12) |  | .61 (*SD* = .30) |  |
| task 5 | old (n = 70) |  | .94 (.08) |  | .80 (.24) |
|  | young (n = 72) |  | .95 (.10) |  | .89 (.14) |
| task 6 | old (n = 70) | .94 (.08) |  | .80 (.24) |  |
|  | young (n = 72) | .91 (.14) |  | .84 (.21) |  |
| task 7 | old (n = 70) |  | .96 (.08) |  | .84 (.16) |
|  | young (n = 72) |  | .95 (.11) |  | .93 (.15) |

*Note:* PR - scores (hits – false alarms) for item- and associative recognition performance for older and younger subjects; Mean (+/-SD). Missing data for 24h delayed recognition and encoding tasks 4-7 from 5 older and 2 younger subjects


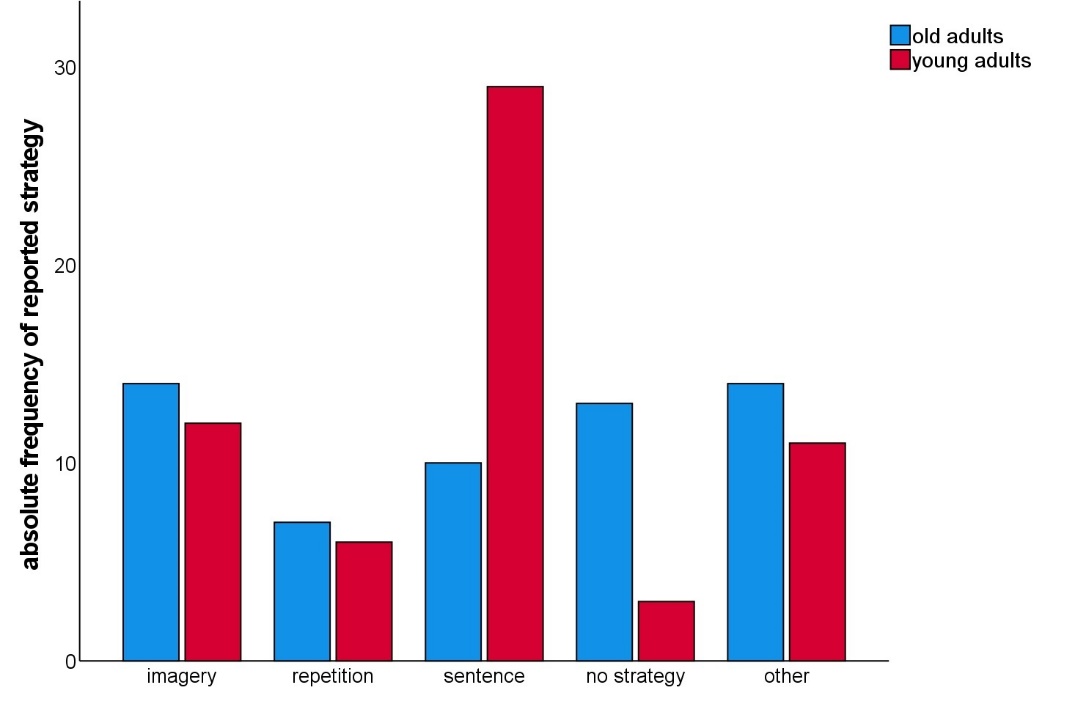


Supplementary Figure 1 Absolute frequency of reported strategies (Task 3). After completing the task, subjects were asked whether they had used a strategy and to describe it. Two separate raters categorized the responses, these categories were also used by Dunlosky & Herzog, 1998. Note: Not all participants completed the debriefing, the analyses are based on: old adults, n = 51 (seven older participants reported using two strategies, these were both counted for each category); young adults, n = 61.
